# Supplementary material for: Self-protection soft fluidic robots with rapid large-area self-healing capabilities
Source: Nat Commun. 2023 Oct 13;14:6430. doi: 10.1038/s41467-023-42214-5 (PMC10576050; doi:10.1038/s41467-023-42214-5)
Supplement: Supplementary file 1 — Supplementary Information [file 41467_2023_42214_MOESM1_ESM.pdf]

Supplementary Information

Self-protection soft fluidic robots with rapid large-area self-healing capabilities

Tang et al.

This PDF file includes:

Supplementary Fig. 1. Design process of a self-protection soft fluidic robot.
Supplementary Fig. 2. Fabrication process of an E-skin.
Supplementary Fig. 3. Fabrication process of a self-protection soft fluidic robot.
Supplementary Fig. 4. Yeoh model fitting for silicone rubber.
Supplementary Fig. 5. Multi-material 3D printing process and printed soft EHD pump.
Supplementary Fig. 6. System actuation speed when a soft EHD pump is embedded into a robot.
Supplementary Fig. 7. Performance tests of a soft EHD pump with two electrode pairs.
Supplementary Fig. 8. Deformation mechanism of the bending soft fluidic robot.
Supplementary Fig. 9. Experimental setups for testing the actuation performances of the self-protection soft fluidic robots.
Supplementary Fig. 10. Static tests of the soft fluidic robots.
Supplementary Fig. 11. Finite element simulation analysis of the bending soft fluidic robot.
Supplementary Fig. 12. Finite element simulation analysis of the twisting soft fluidic robot.
Supplementary Fig. 13. Finite element simulation analysis of the contracting soft fluidic robot.
Supplementary Fig. 14. PID closed-loop control system.
Supplementary Fig. 15. Self-sensing of self-protection soft fluidic robots.
Supplementary Fig. 16. Tensile tests.
Supplementary Fig. 17. The heating process curve of the E-skin.
Supplementary Fig. 18. Untethered soft gripper.
Supplementary Fig. 19. Actuators of the mechanical sieve.
Supplementary Table 1. Comparison of different self-healing of soft materials.
Supplementary Table 2. Comparison of different soft fluidic systems.
Supplementary Table 3. System integration level of demonstrations.
Supplementary References

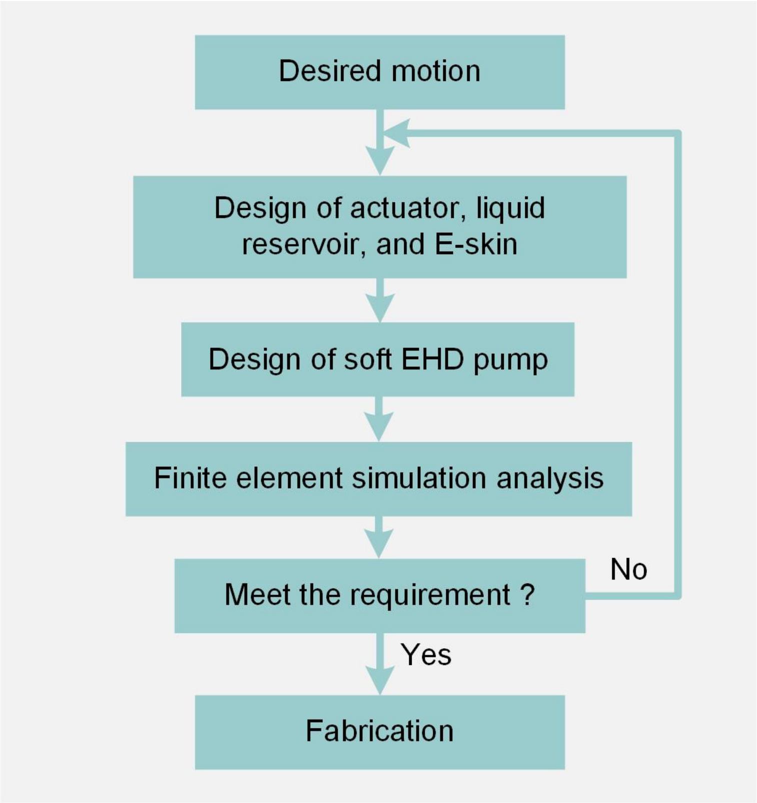

Supplementary Fig. 1 | Design process of a self-protection soft fluidic robot. This design process allows for the development of self-protection soft fluidic robots for various motions.

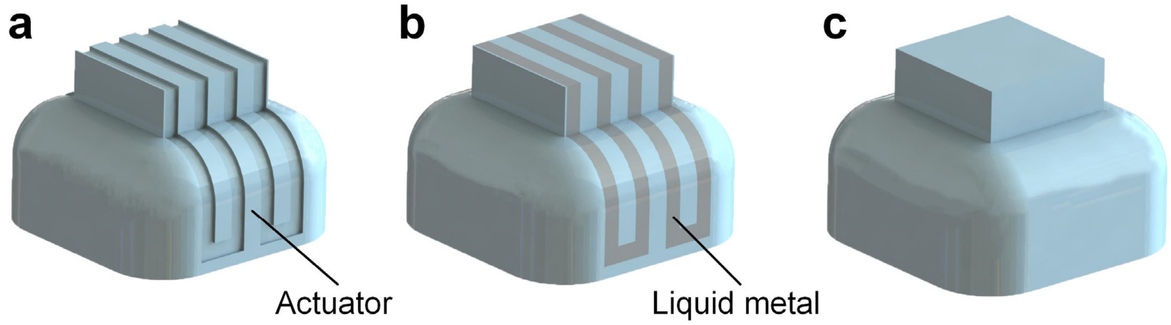

Supplementary Fig. 2 | Fabrication process of an E-skin. **a**, Actuator. **b**, E-skin without a seal. **c**, E-skin with a seal.

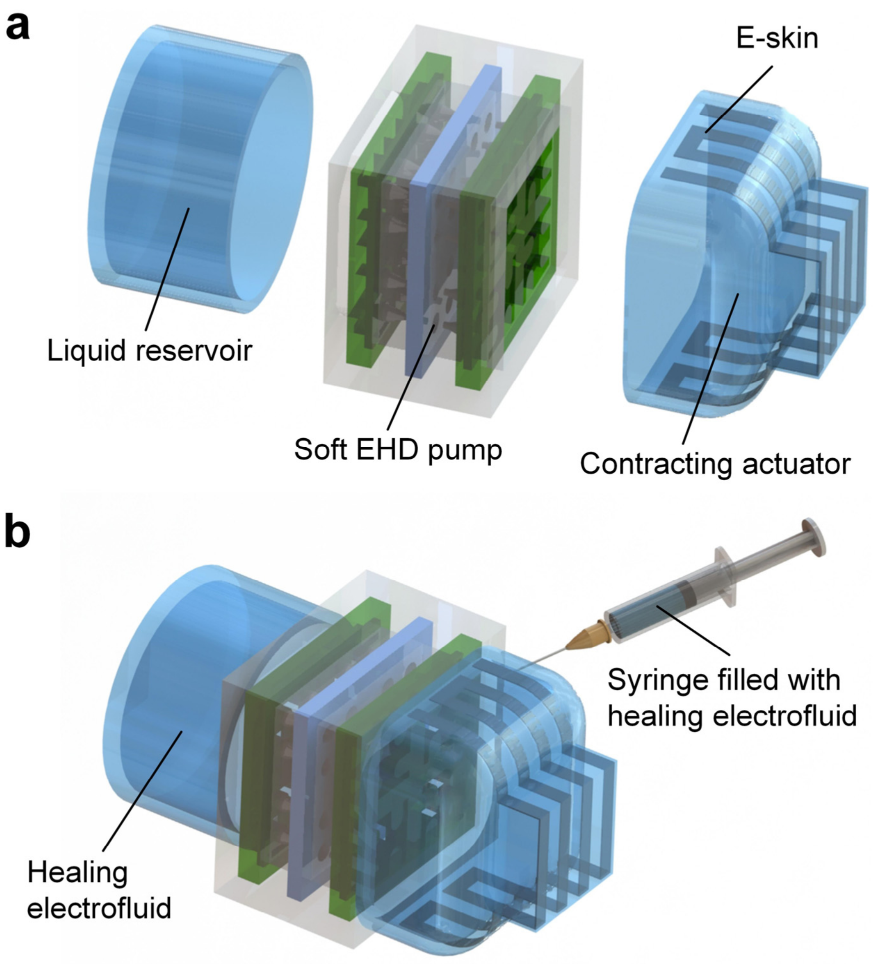

Supplementary Fig. 3 | Fabrication process of a self-protection soft fluidic robot. a, A contracting actuator, a soft EHD pump, and a liquid reservoir are bonded to form a soft structure with two chambers. **b**, Uncured white Ecoflex 00-20 is poured into the surface of the soft EHD pump to achieve better sealing and an appropriate amount of healing electrofluid is filled into the chambers by using a syringe with a needle. The adhesive is applied on the punctured area of the needle to entirely seal the chambers.

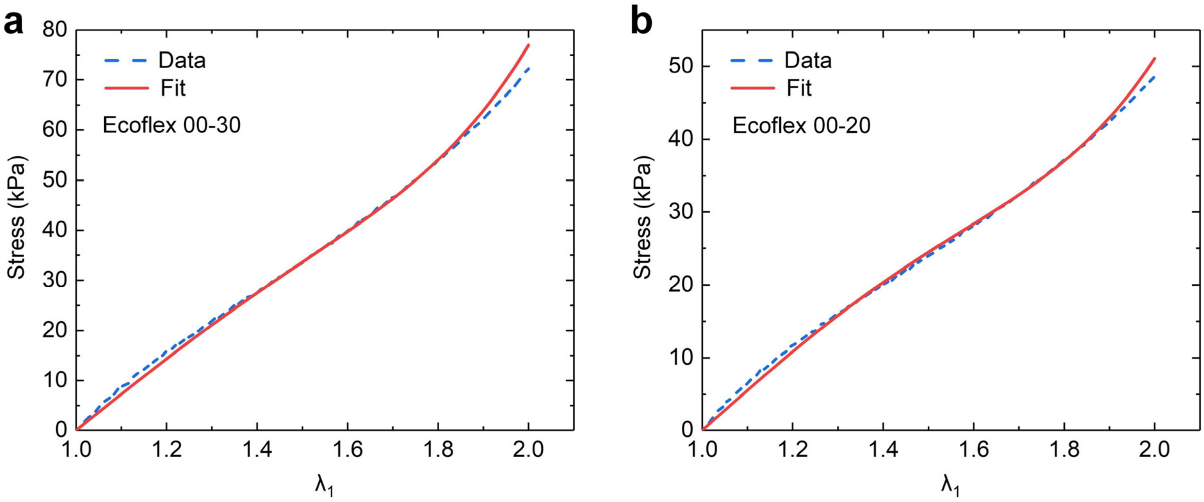

Supplementary Fig. 4 | Yeoh model fitting for silicone rubber. a, Ecoflex 00-30. **b**, Ecoflex 00-20.

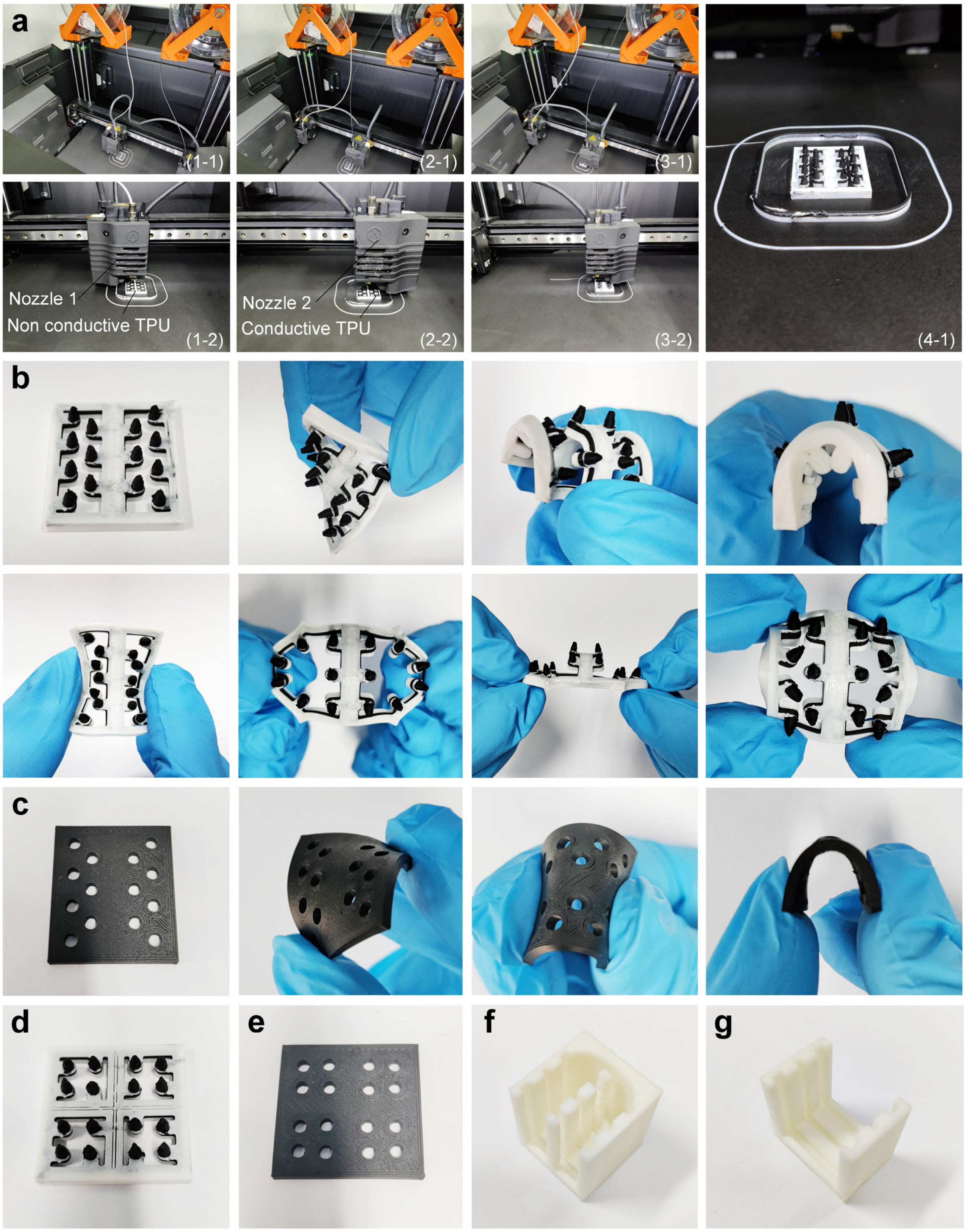

Supplementary Fig. 5 | Multi-material 3D printing process and printed soft EHD pump. **a**, Multi-material 3D printing process. **b**, Printed conical array electrodes. **c**, Printed porous plate electrodes, 27 mm × 23 mm × 2 mm. **d**, Printed conical array electrodes. **e**, Printed porous plate electrodes, 26 mm × 26 mm × 2 mm. **f**, Printed slots. **g**, Printed slots.

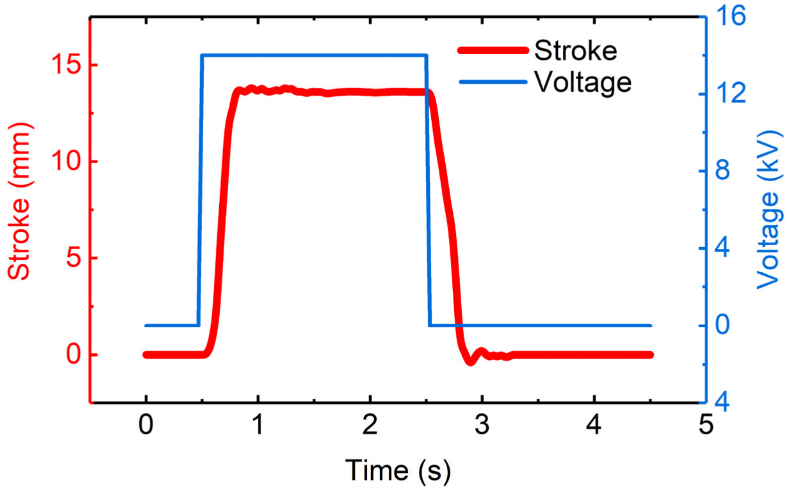

Supplementary Fig. 6 | System actuation speed when a soft EHD pump is embedded into a robot. The robot is a contracting fluidic robot with a 20-g load. It takes ~ 0.25 s for the robot to reach the maximum stroke of 14 mm. Response time for other deformations is less than 0.25 s.

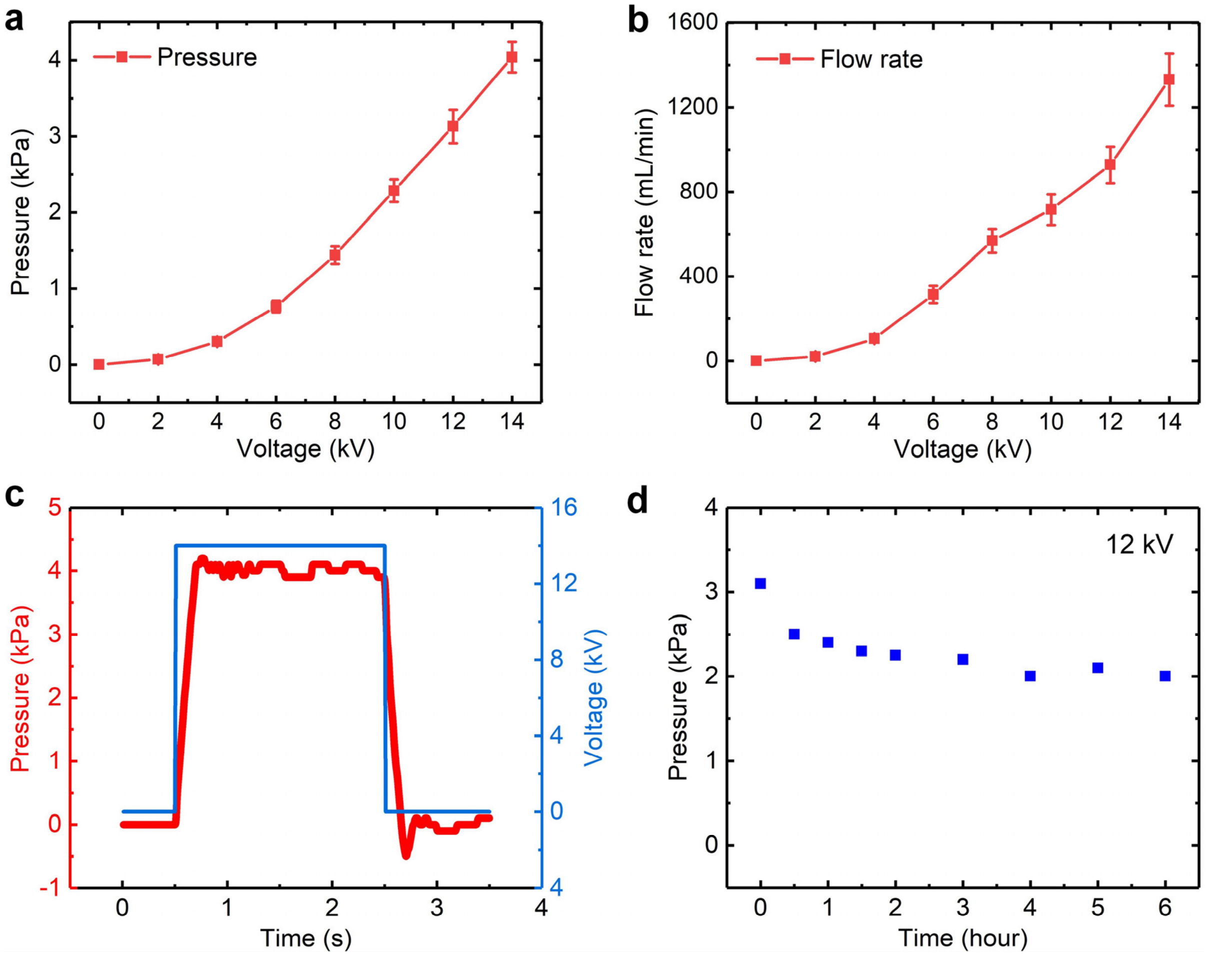

Supplementary Fig. 7 | Performance tests of a soft EHD pump with two electrode pairs. **a**, Pressure-voltage curve. **b**, Flow rate-voltage curve. **c**, Response time curve. The response time (peak time) of the soft EHD pump is ~ 0.2 s. **d**, Lifetime test of the soft EHD pump. We test the lifetime of the soft EHD pumps at an applied voltage of 12 kV for 6 h, illustrating the durability and reliability of the soft EHD pumps. It is worth mentioning that the electrodes would be passivated and then stabilized when a high voltage was supplied for a longer period of time, causing the pressure to drop and then stable[17].

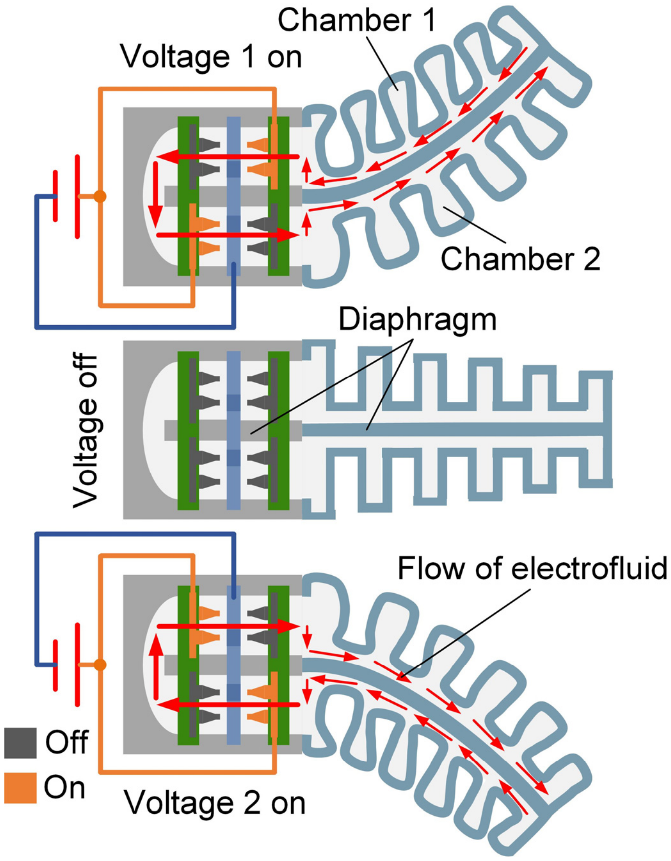

Supplementary Fig. 8 | Deformation mechanism of the bending soft fluidic robot. When voltage 1 is on and voltage 2 is off, the two electrode pairs on the soft EHD pump operate to pump the electrofluid from chamber 1 to chamber 2, resulting in an upward bending motion of the robot. Conversely, when voltage 2 is on and voltage 1 is off, the other two electrode pairs on the soft EHD pump work to pump the electrofluid from chamber 2 to chamber 1, resulting in a downward bending motion of the robot. When both voltages are off, the electrofluid flows back to its original location and the robot returns to its initial state.

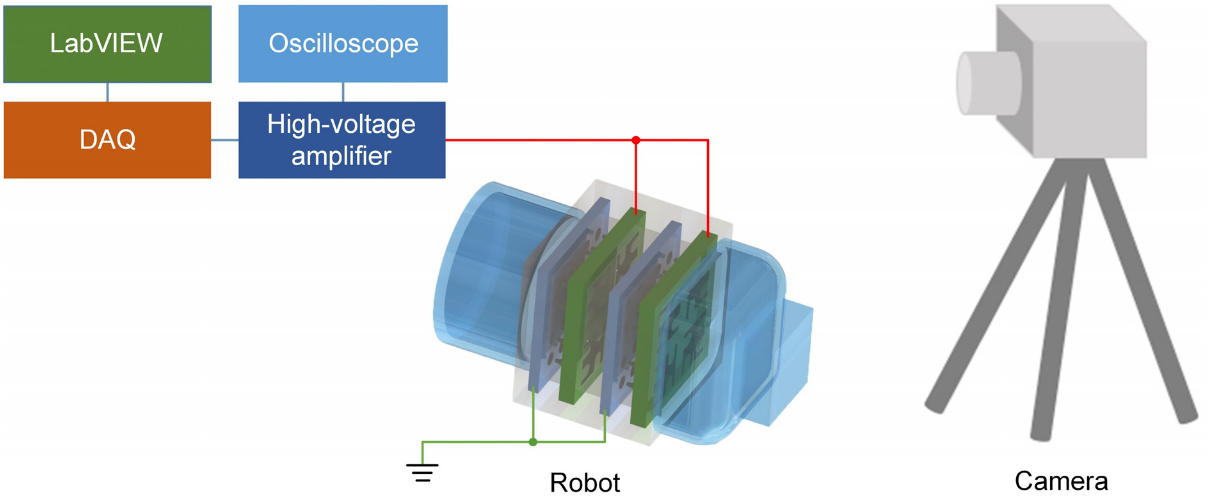

Supplementary Fig. 9 | Experimental setups for testing the actuation performances of the self-protection soft fluidic robots.

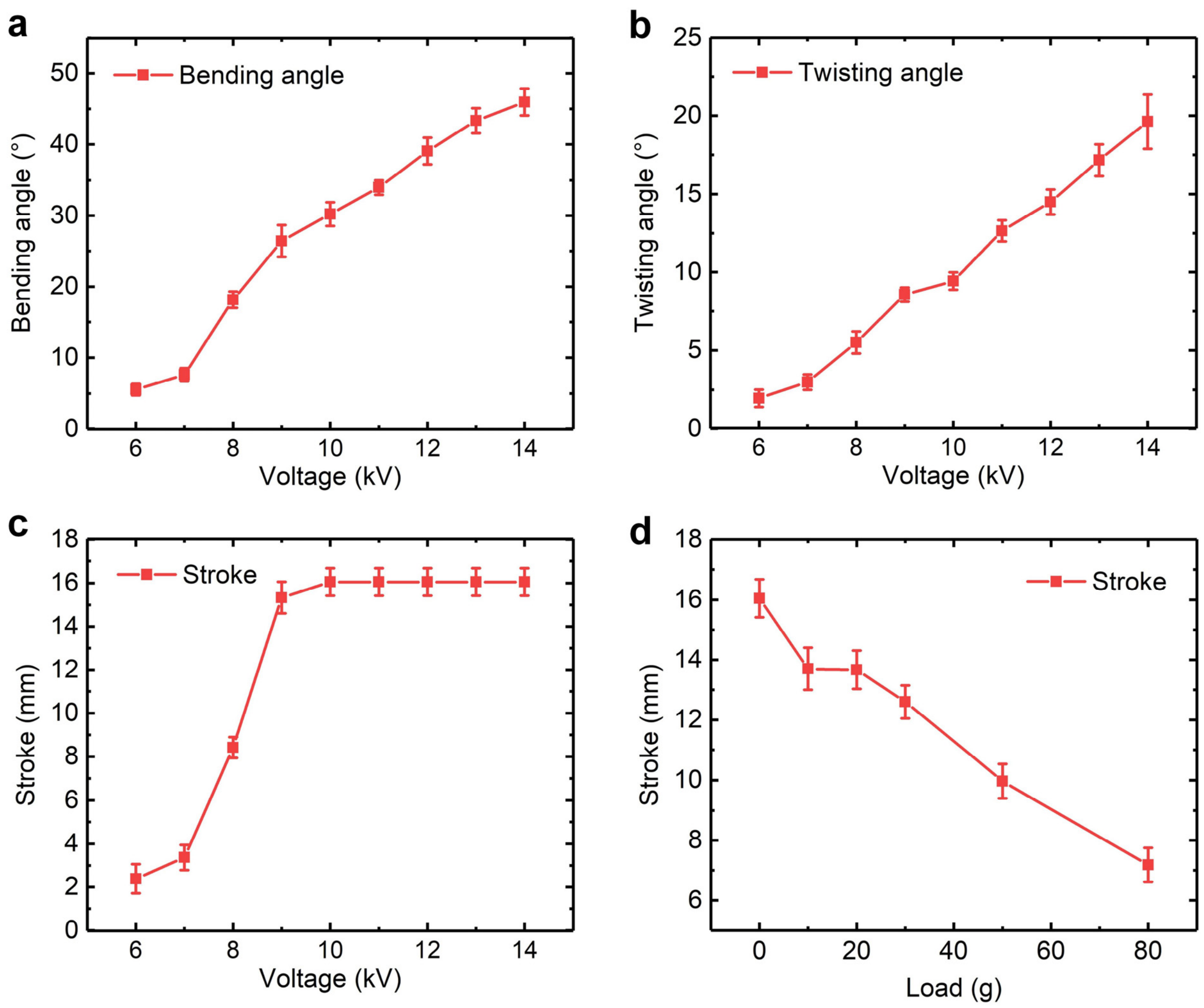

Supplementary Fig. 10 | Static tests of the soft fluidic robots. a, Bending angle – voltage curve of the bending fluidic robot (unilateral bending). **b,** Twisting angle – voltage curve of the twisting fluidic robot. **c,** Actuation stroke – voltage curve of the contracting fluidic robot. **d,** Actuation stroke – load curve of the contracting fluidic robot. The applied voltage is 14 kV.

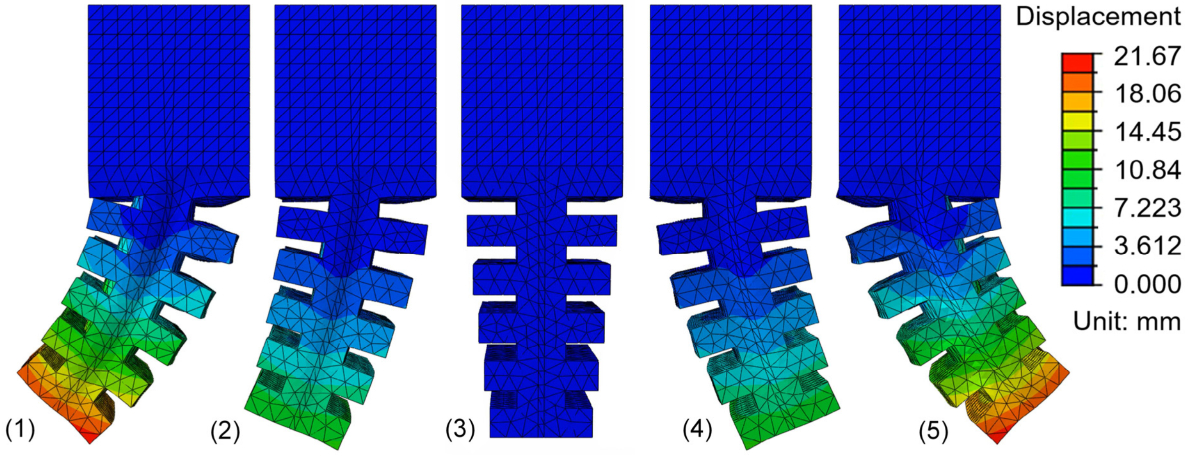

Supplementary Fig. 11 | Finite element simulation analysis of the bending soft fluidic robot. (3)-(1) are the processes of bending to the left side, (1)-(5) are the processes of bending to the right side.

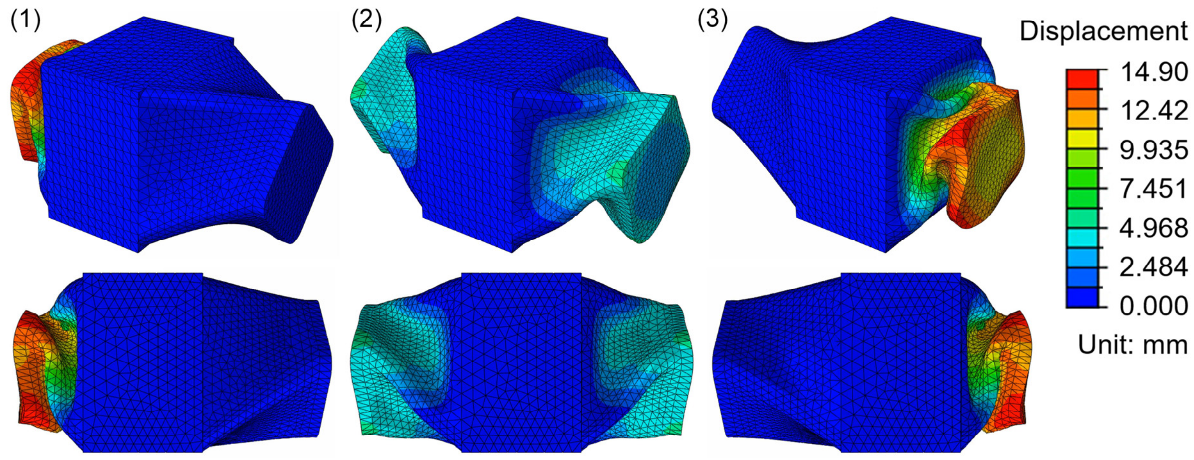

Supplementary Fig. 12 | Finite element simulation analysis of the twisting soft fluidic robot. The simulation results are a deformation effect of two chambers superimposed on each other, where two chambers twist in different directions.

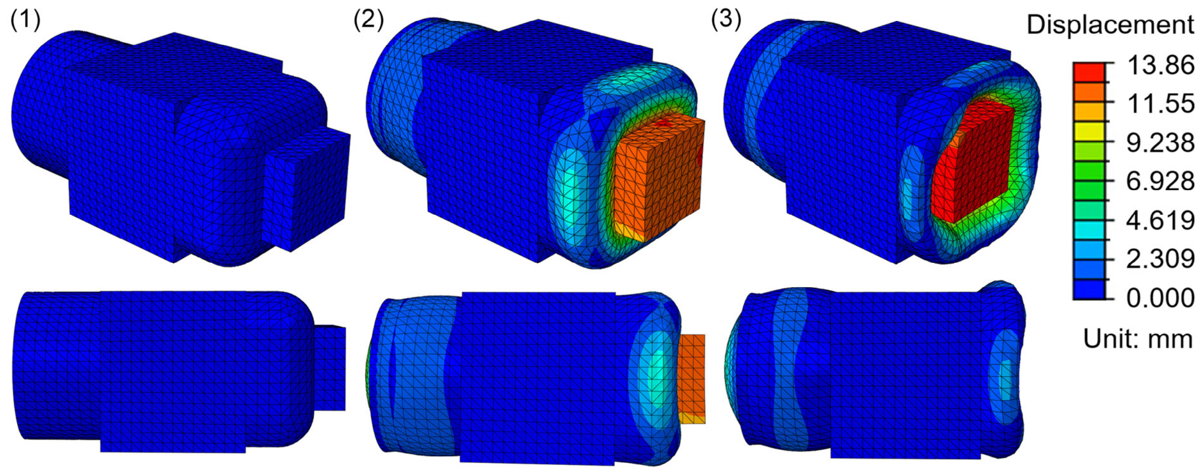

Supplementary Fig. 13 | Finite element simulation analysis of the contracting soft fluidic robot. The actuator produces a contracting motion while the liquid reservoir expands.

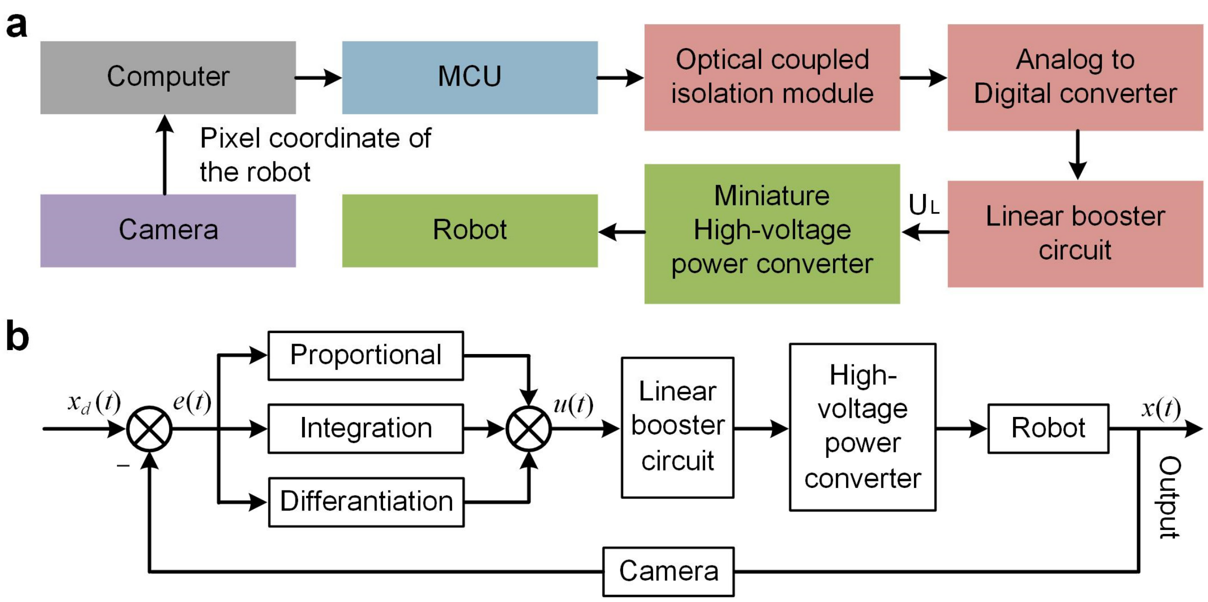

Supplementary Fig. 14 | PID closed-loop control system. a, Hardware of the system. **b**, PID control principle of the robot.

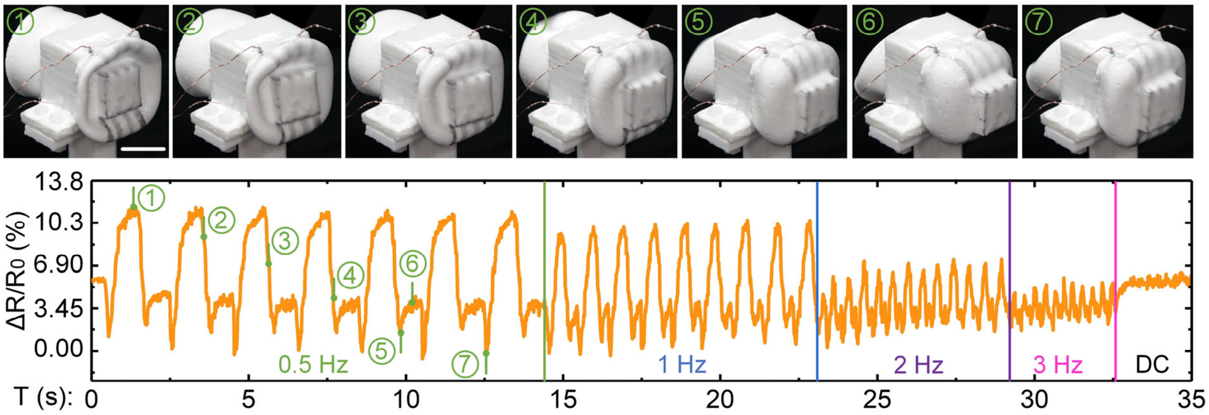

Supplementary Fig. 15 | Self-sensing of self-protection soft fluidic robots. The amplitude of the voltage is 14 kV, and the frequencies range from 0.5 Hz to 3 Hz. The curves of the resistance value output from the E-skin are consistent and stable for the same actuation frequency, indicating that the E-skin can respond well to the deformation of the robot during the actuation process. The scale bar is 2 cm.

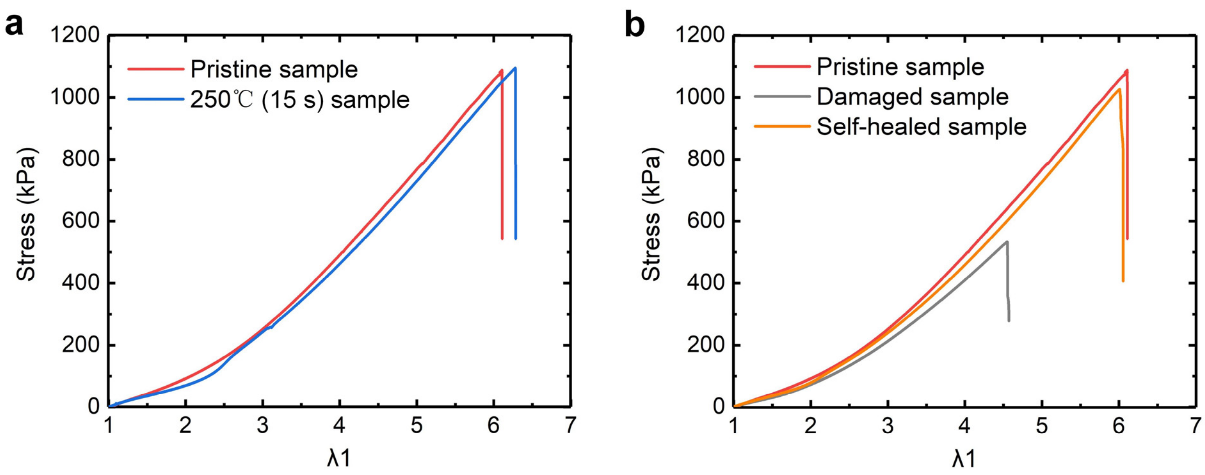

Supplementary Fig. 16 | Tensile tests. To test the mechanical properties of samples (silicone 5A), uniaxial tensile tests were conducted at an ASTM D638 (Type IV) universal test machine with a crosshead speed of 50 mm/min. **a**, Tensile stress-strain curves of pristine and 250 °C (15 s) samples. **b**, Tensile stress-strain curves of pristine, damaged, and self-healed samples.

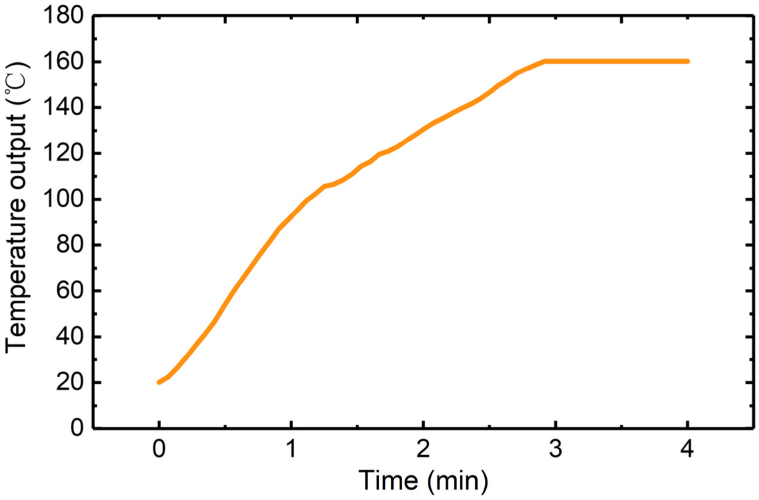

Supplementary Fig. 17 | The heating process curve of the E-skin. The temperature gradually increases with the heating time and rises to ~ 160 °C in 3 minutes, after which the temperature of the E-skin stabilizes at ~ 160 °C. The input power of the E-skin is ~ 4 W.

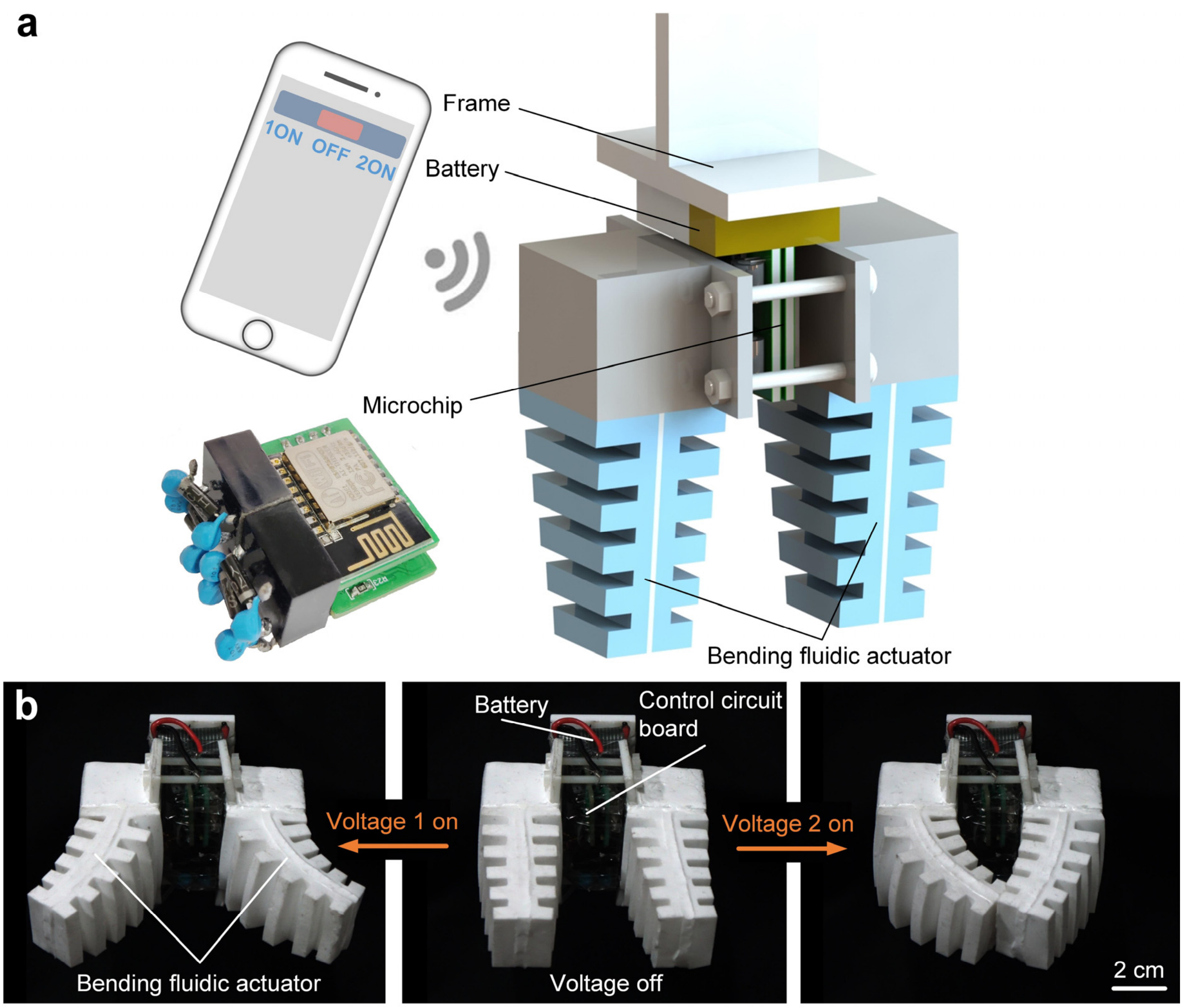

Supplementary Fig. 18 | Untethered soft gripper. a, Architecture of the soft gripper. The soft gripper consists of two bending fluidic actuators, a microchip, a battery and a frame. **b**, Actuation process of the soft gripper. The gripper opens outward when voltage 1 is on, closes when voltage 2 is on, and resets when power is off.

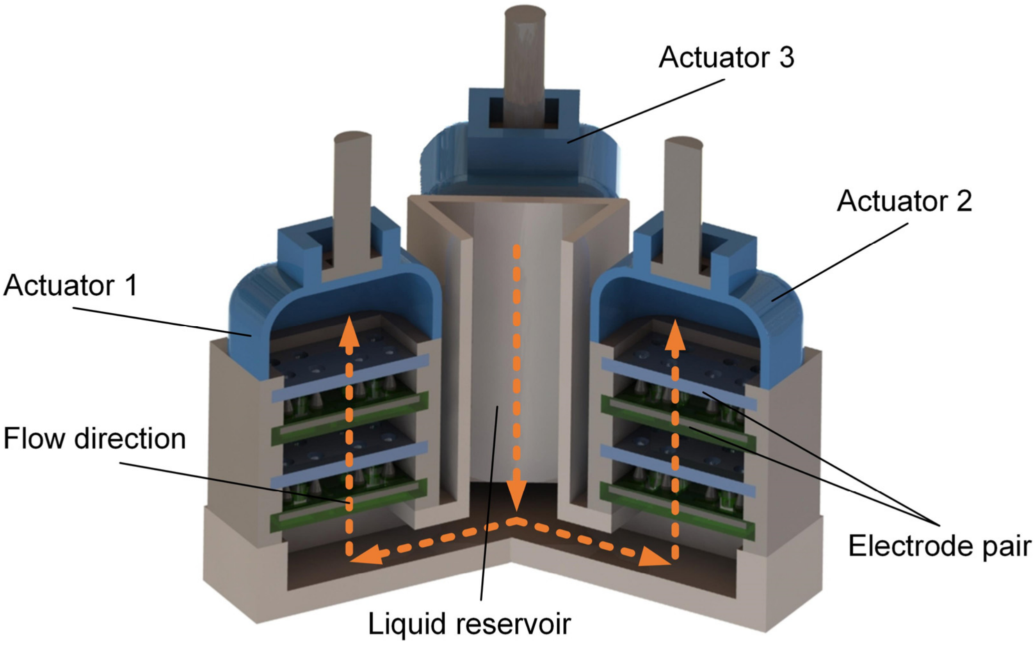

Supplementary Fig. 19 | Actuators of the mechanical sieve. The fluidic robot consists of three actuators, several electrode pairs, and a reservoir cylinder. Three actuators are integrated in a fixed frame and share the middle reservoir cylinder, and the three actuators and the bottom of the reservoir cylinder are connected.

Supplementary Table 1 | Comparison of different self-healing of soft materials.

	Type of damage and stretch rate	Time and temperature
Diels-Alder polymer[21]	Only small-area, non-autonomous, ~ 100%	> 24 h, 80 °C
Photopolymerizable self-healing[23]	Only small-area, non-autonomous, ~ 1000%	> 24 h, 90 °C
Self-healing polyurethane urea elastomer[25]	Only small-area, autonomous, ~ 1100%	~ 6 h, 25 °C
Diels-Alder polymer[27]	Only small-area, autonomous, < 100%	> 24 h, 90 °C
Printed Diels-Alder polymer[30]	Only small-area, non-autonomous, ~ 100%	> 24 h, 120 °C
Healing agents[31]	Only small-area, autonomous, non-stretchable	> 48 h, 25 °C
Healing liquid[17]	Only small-area, autonomous, ~ 20%	> 6 h, 35 °C
Healing electrofluid (This study)	Large-area, autonomous, > 1200%	10 s, 250 °C

Supplementary Table 2 | Comparison of different soft fluidic systems.

	Design	Self-contained	Frequency	Self-healing	Intelligent level
Fluid-powered Fiber-reinforced soft actuators[34]	Diverse	No	1-5 Hz	No	None
Fluid-driven origami-inspired artificial muscles[35]	Diverse	No	1-5 Hz	No	None
STAUD-prepreg soft fluidic actuators[36]	Diverse	No	1-5 Hz	No	None
Soft fluidic actuators with programmable bioinspired architectures[37]	Diverse	No	1-5 Hz	No	None
3D-printed soft fluidic actuators[38]	Linear	No	1-5 Hz	No	None
Untethered soft fluidic robot[39]	Bend	Yes	< 1 Hz	No	None
Electro-pneumatic pump driven soft fluidic actuators[40]	Contract	Yes	~ 5 Hz	No	None
Gas-liquid phase change powered pneumatic artificial muscles[10]	Diverse	Yes	< 0.1 Hz	No	None
Electro-conjugate fluid pump driven fluidic flexible finger[15]	Bend	Yes	~ 1 Hz	No	None
Stretchable pump driven bending fluidic actuator[16]	Bend	Yes	< 0.1 Hz	No	None
Self-contained soft electrofluidic actuators[19]	Linear, bend	Yes	~ 30 Hz	No	None
Self-healing soft pneumatic robots[21]	Diverse	No	1-5 Hz	Small-area	None
Self-sensing soft fluidic actuators[26]	Diverse	No	1-5 Hz	No	Self-sensing
Healable resistive heater in self-healing soft robots[27]	Bend	No	1-5 Hz	Small-area	Self-heating
Damage intelligent soft robots[25]	Bend	No	1-5 Hz	Small-area	Self-detecting
Self-protection soft fluidic robots (This study)	Diverse	Yes	~ 50 Hz	Large-area	Self-sensing, self-judgment, self-heating for rapid self-healing

Supplementary Table 3 | System integration level of demonstrations.

	System integration level
Supplementary Movie 1	Full integrated system (untethered)
Supplementary Movie 2	Full integrated system (untethered)
Supplementary Movie 3	Full integrated system (untethered)
Supplementary Movie 4	Tethered
Supplementary Movie 5	Tethered
Supplementary Movie 6	Full integrated system (untethered)
Supplementary Movie 7	Tethered
Supplementary Movie 8	Full integrated system (untethered)
Supplementary Movie 9	Full integrated system (untethered)

Supplementary References (a part of the main text references)

10. Zhong, Y. et al. Programmable thermochromic soft actuators with “two dimensional” bilayer architectures for soft robotics. *Nano Energy* **102**, 107741 (2022).
15. Nagaoka, T., Mao, Z., Takemura, K., Yokota, S. & Kim, J.-w. ECF (electro-conjugate fluid) finger with bidirectional motion and its application to a flexible hand. *Smart Mater. Struct.* **28**, 025032 (12pp) (2019).
16. Cacucciolo, V. et al. Stretchable pumps for soft machines. *Nature* **572**, 516–519 (2019).
17. Tang, W. et al. Customizing a self-healing soft pump for robot. *Nat. Commun.* **12**, 2247 (2021).
19. Tang, W. et al. Self-contained soft electrofluidic actuators. *Sci. Adv.* **7**, eabf8080 (2021).
21. Terryn, S., Brancart, J., Lefeber, D., Assche, G. V. & Vanderborght, B. Self-healing soft pneumatic robots. *Sci. Robot.* **2**, eaan4268 (2017).
23. Gomez, E. F. et al. 3D-Printed Self-Healing Elastomers for Modular Soft Robotics. *ACS Appl. Mater. Interfaces* **13**, 28870−28877 (2021).
25. Bai, H., Kim, Y. S. & Shepherd, R. F. Autonomous self-healing optical sensors for damage intelligent soft-bodied systems. *Sci. Adv.* **8**, eabq2104 (2022).
26. Kim, S. Y. et al. Sustainable manufacturing of sensors onto soft systems using self-coagulating conductive Pickering emulsions. *Sci. Robot.* **5**, eaay3604 (2020).
27. Tabrizian, S. K. et al. A healable resistive heater as a stimuli-providing system in self-healing soft robots. *IEEE Robot. Autom. Lett.* **7**, 4574–4581 (2022).
30. Roels, E. et al. Additive Manufacturing for Self-Healing Soft Robots. *Soft Robot.* **7**, 711-723 (2020).
31. Cuvellier, A., Torre-Muruzabal, A., Van Assche, G., Clerck, K. De & Rahier, H. Selection of healing agents for a vascular self-healing application. *Polym. Test.* **62**, 302-310 (2017).
34. Connolly, F., Walsh, C. J. & Bertoldi, K. Automatic design of fiber-reinforced soft actuators for trajectory matching. *Proc. Natl. Acad. Sci. U.S.A.* **114**, 51–56 (2017).
35. Li, S., Vogt, D. M., Rus, D. & Wood, R. J. Fluid-driven origami-inspired artificial muscles. *Proc. Natl. Acad. Sci. U.S.A.* **114**, 13132–13137 (2017).
36. Kim, S. Y. et al. Reconfigurable soft body trajectories using unidirectionally stretchable composite laminae. *Nat. Commun.* **10**, 3464 (2019).
37. Schaffner, M. et al. 3D printing of robotic soft actuators with programmable bioinspired architectures. *Nat. Commun.* **9**, 878 (2018).
38. Pascali, C. De, Naselli, G. A., Palagi, S., Scharff, R. B. N. & Mazzolai, B. 3D-printed biomimetic artificial muscles using soft actuators that contract and elongate. *Sci. Robot.* **7**, eabn4155 (2022).
39. Tolley, M. T. et al. A Resilient, Untethered Soft Robot. *Soft Robot.* **1**, 213-223 (2014).
40. Diteesawat, R. S., Helps, T., Taghavi, M. &Rossiter, J. Electro-pneumatic pumps for soft robotics. *Sci. Robot.* **6**, eabc3721 (2021).
