# Supplementary material for: Self-protection soft fluidic robots with rapid large-area self-healing capabilities
Source: Nat Commun. 2023 Oct 13;14:6430. doi: 10.1038/s41467-023-42214-5 (PMC10576050; doi:10.1038/s41467-023-42214-5)
Supplement: Supplementary file 3 — Description of Additional Supplementary Files [file 41467_2023_42214_MOESM3_ESM.pdf]

Description of Additional Supplementary Files

Supplementary Movie 1: High-speed actuation of bending soft fluidic robots.

Supplementary Movie 2: High-speed actuation of twisting soft fluidic robots.

Supplementary Movie 3: High-speed actuation of contracting soft fluidic robots.

Supplementary Movie 4: Frequency response of contracting soft fluidic robots.

Supplementary Movie 5: Self-sensing of contracting soft fluidic robots under different frequencies.

Supplementary Movie 6: Self-sensing, self-judgment, self-heating and self-healing of soft fluidic robots.

Supplementary Movie 7: Increasing forces.

Supplementary Movie 8: An untethered soft gripper.

Supplementary Movie 9: A mechanical sieve.
